# DDX56 inhibits type I interferon by disrupting assembly of IRF3–IPO5 to inhibit IRF3 nucleus import

**DOI:** 10.1242/jcs.230409

**Published:** 2019-07-24

**Authors:** Dan Li, Shaozu Fu, Zhengqian Wu, Wenping Yang, Yi Ru, Hongbing Shu, Xiangtao Liu, Haixue Zheng

**Affiliations:** 1 State Key Laboratory of Veterinary Etiological Biology and OIE/National Foot and Mouth Disease Reference Laboratory, Lanzhou Veterinary Research Institute, Chinese Academy of Agricultural Sciences, Lanzhou, Gansu 730046, China; 2 Medical Research Institute, Wuhan University, Wuhan 430072, China

**Keywords:** DDX56, Type I interferon, IRF3, IPO5, Nucleus import

## Abstract

Transcription factor IRF3-mediated type I interferon induction plays a role in antiviral innate immunity. However, mechanisms for the control and regulation of IRF3 nuclear import remain largely unknown. We have identified DEAD box polypeptide 56 (DDX56) as a negative regulator of virus-triggered IFN-β induction. Overexpression of DDX56 suppressed nuclear translocation of IRF3 via disrupting the IRF3–IOP5 interaction, whereas knockdown or knockout of DDX56 had the opposite effect. In addition, the interaction between DDX56 and IRF3 increased during viral infection. We further found that the D166 site of DDX56 was essential for inhibiting IRF3 import into the nucleus. Our findings suggest that DDX56 regulates antiviral innate immunity by inhibiting the nuclear translocation of IRF3, revealing a novel mechanism of the DDX56-mediated innate antiviral response.

This article has an associated First Person interview with the first author of the paper.

## Abstract (precis)

**Summary:** DDX56 is a negative regulator of virus-triggered IFN-β induction that acts by disruputing the IRF3–IOP5 interaction to inhibit the import of IRF3 into the nucleus.

## INTRODUCTION

To defend against invading viruses, the innate immune response is initiated through a variety of pattern-recognition receptors in the host that recognize pathogen-associated molecular patterns (Janeway, 1989). For example, the RIG-I-like receptors, including RIG-I (also known as DDX58) and MDA5 (also known as IFIH1), recognize viral double-stranded RNA or single-stranded RNA in most cells (Akira et al., 2006) through their C-terminal RNA helicase domains. They then undergo conformational changes and are recruited to the downstream adaptor protein VISA (also known as MAVS, IPS-1 and Cardif) through their CARD domains (O'Neill and Bowie, 2010; Xu et al., 2005). Membrane-associated Toll-like receptors (TLRs), such as TLR3, TLR7, TLR8 and TLR9, detect virus DNA or RNA in the endosome and trigger TIR domain-containing adaptor-inducing interferon (IFN)-β (TRIF)- and MYD88-mediated signaling pathways (Barbalat et al., 2011; Takeuchi and Akira, 2010). The activation of pattern-recognition receptors triggers a series of downstream cellular events that lead to the rapid production of type I interferons and proinflammatory cytokines (Akira et al., 2006; Takeuchi and Akira, 2010).

The interferon-regulatory factor 3 (IRF3) is a master transcription factor responsible for the induction of the type I interferons and is essential for the host antiviral innate immune responses (Honda and Taniguchi, 2006). Upon virus infection, IRF3 is phosphorylated by the kinases TBK1 and IKKε at its C-terminus, forming dimers that are then transported into the nucleus (Honda and Taniguchi, 2006; Zhao et al., 2007), subsequently forming a complex with the coactivators of the p300/CBP family and initiating transcription of target genes, including IFN-β (Servant et al., 2003; Takahasi et al., 2010). Furthermore, phosphorylation of IRF3 is an indispensable step for its activation, and phosphorylated IRF3 is translocated into the nucleus to bind the IFN-β (*IFNB1*) promoter (Song et al., 2016). Previous studies have shown that IRF3 has an active nuclear localization signal that is recognized by importin-α receptors and is transported into the nucleus (Kumar et al., 2000; Zhu et al., 2015) by an active nuclear export signal that is exported from the nucleus via the CRM1-mediated pathway. This signal mainly localizes in the cytoplasm in unstimulated cells (Yoneyama et al., 1998). The activated IRF3 resides in the nucleus and interacts with CBP (also known as CREBBP) (Kumar et al., 2000; Lin et al., 1998). However, the mechanism underlying the IRF3 translocation process remains elusive.

In this study, we identified DDX56 (also known as DDX21, DDX26 or NOH61) as a negative regulator of virus-triggered type I IFN induction. We further found that DDX56 acts by disrupting the interaction between IRF3 and IPO5, which inhibits the nuclear translocation of IRF3. Our study demonstrates a novel mechanism through which DDX56 regulates virus-triggered IFN induction.

## RESULTS

### Overexpression of DDX56 inhibits virus-triggered activation of IFN-β

**Fig. 1.**
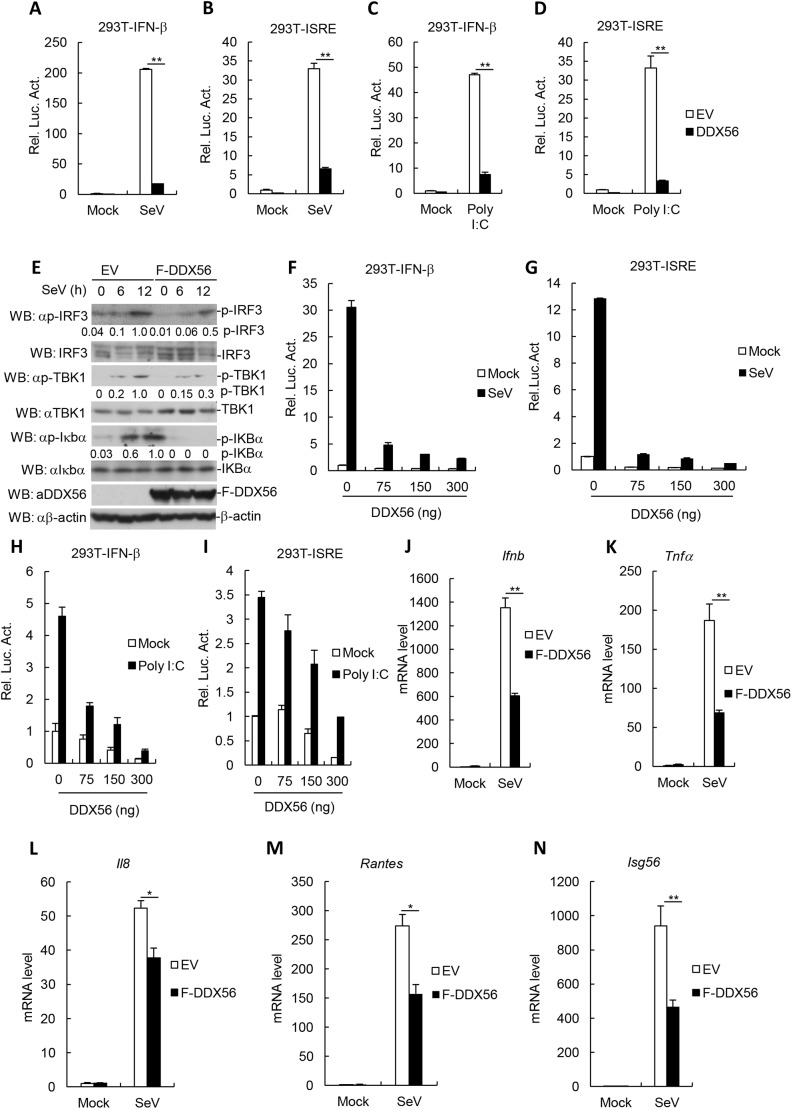
**The overexpression of DDX56 markedly inhibits the virus-triggered IFN-β signaling pathway.** (A–D) DDX56 inhibited the SeV- or poly(I:C)-induced activations of the ISRE and the IFN-β promoter in 293T cells. 293T cells (10^5^) were transfected with the ISRE or IFN-β promoter luciferase plasmids (0.1 μg) and the DDX56 expression plasmid (100 ng). At 20 h after transfection, the cells were infected with or without SeV for 10 h or were treated with poly(I:C) (1 µg/ml) or left untreated for 18 h before the luciferase assays were performed. Shown are representative experiments of three independent experiments with mean±s.d. of three technical replicates. **P<0.01. (E) Overexpression of DDX56 inhibits the SeV-induced phosphorylation of TBK1, IRF3 and IκBα. The stably transfected DDX56 293T cells were infected with SeV for the indicated times. Cell lysates were analyzed by immunoblotting (WB) with the indicated antibodies. The degree of protein phosphorylation was calculated by ImageJ software and is presented under the blot. (F–I) DDX56 inhibited the SeV- and poly(I:C)-induced activations of ISRE and the IFN-β promoter in a dose-dependent manner in 293T cells. 293T cells (10^5^) were transfected with the ISRE or IFN-β promoter luciferase plasmids (0.1 μg) and the indicated amount of DDX56 expression plasmid. At 24 h after transfection, the cells were infected with or without SeV for 10 h or were treated with poly(I:C) (1 µg/ml) or left untreated for 18 h before the luciferase assays were performed. Shown are representative experiments of three independent experiments with the mean±s.d. of three technical replicates. (J–N) Effects of DDX56 overexpression on SeV-triggered transcription of *IFNB1*, *TNFa*, *Il8*, *Rantes* and *Isg56* genes. The DDX56-overexpressing stable 293T cells (4×10^5^) were left uninfected or infected with SeV for 12 h before qRT-PCR was performed. Shown are representative experiments of three independent experiments with the mean±s.d. of three technical replicates. *P<0.05, **P<0.01. EV, empty vector; Luc, luciferase; F, Flag tag.

Because DDX56 is important for infectivity of West Nile virus (WNV) virions, and is required for the assembly of infectious WNV virions (Reid and Hobman, 2017; Xu et al., 2011), we examined whether DDX56 is involved in the regulation of the virus-triggered IFN-β signaling pathway. In reporter assays, overexpression of DDX56 significantly inhibited Sendai virus (SeV) or polyinosinic:polycytidylic acid [poly(I:C)]-triggered activation of the IFN-β promoter and interferon-sensitive response element (ISRE) in human embryonic 293T cells (Fig. 1A–D). As shown in Fig. 1E, SeV-triggered phosphorylation of TBK1, IRF3 and IkBa (also known as NFKBIA), which are hallmarks of the activation of virus-triggered IFN induction pathways, were markedly lower in DDX56-overexpressing cells in comparison to in control cells. Further experiments indicated that the overexpression of DDX56 inhibited SeV- and the poly(I:C)-triggered activation of the IFN-β promoter and ISRE in a dose-dependent manner in 293T cells (Fig. 1F–I). In addition, we observed that SeV-triggered transcription of the *Ifnb1*, *Tnfa*, *Il8*, *Rantes* (also known as *Ccl5*) and *Isg56* genes was markedly inhibited in DDX56-overexpressing 293T and THP-1 cells in comparison with control cells (Fig. 1J–N; Fig. S1A–E). In addition, we performed an MTT assay and flow cytometry analysis to confirm effects of DDX56 on the viability and apoptosis of cells. The flow cytometry data demonstrated that the rate of cell apoptosis was not significant different in cells overexpressing DDX56 compared with that for the control cells (Fig. S1F). Similarly, the MTT assay experiments indicated that overexpressing DDX56 did not significantly affect cell viability in comparison to that for control cells (Fig. S1G). These results suggest that DDX56 inhibits virus-triggered activation of the IFN-β signaling pathway and does not affect the viability and apoptosis of cells.

### Knockdown of DDX56 potentiates virus-triggered induction of IFN-β

**Fig. 2.**
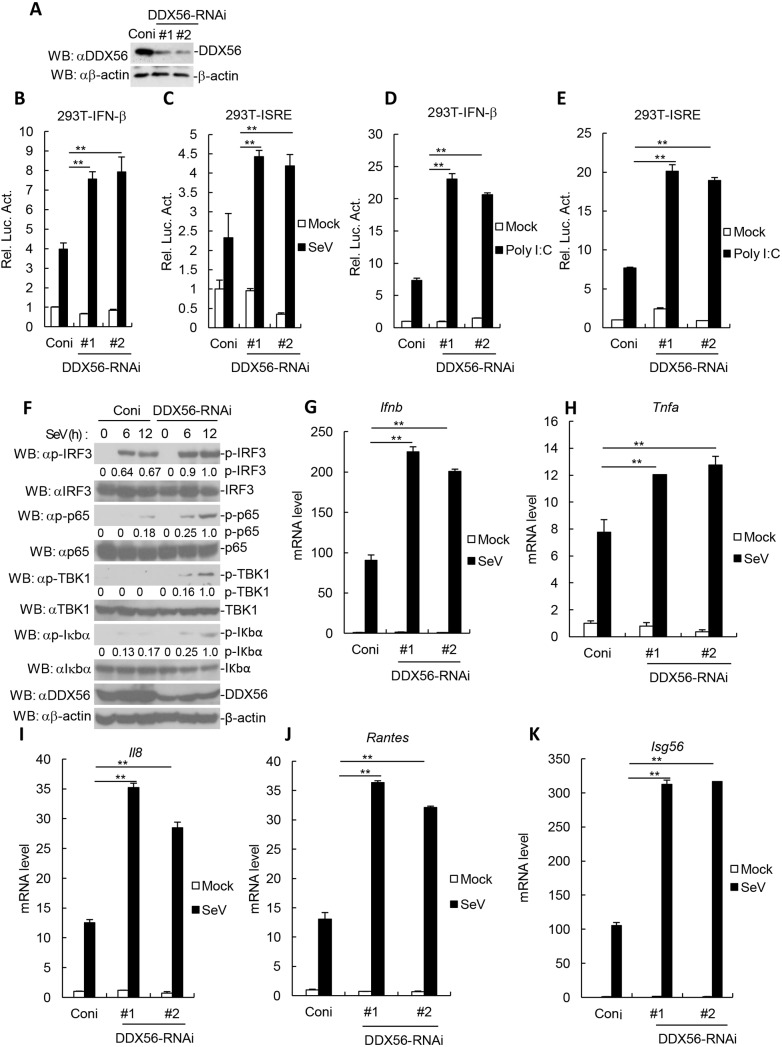
**Knockdown of DDX56 potentiates RNA virus-triggered signaling.** (A) Effects of DDX56-RNAi plasmids on the expression of endogenous DDX56. (B–E) Effects of DDX56-RNAi plasmids on SeV or poly(I:C)-triggered activation of the IFN-β promoter and ISRE. Stable DDX56-knockdown 293T cells (10^5^) were transfected with the IFN-β promoter or ISRE (100 ng). At 24 h after transfection, the cells were left uninfected or infected with SeV for 12 h, or were treated with poly(I:C) (1 µg/ml) or left untreated for 18 h before reporter assays were performed. Shown are representative experiments of three independent experiments with mean±s.d. of three technical replicates. **P<0.01. (F) Knockdown of DDX56 increases the SeV-induced phosphorylation of TBK1, IRF3 and IκBα. The stable DDX56-knockdown 293T cells were infected with SeV for the indicated times. Cell lysates were analyzed by immunoblotting (WB) with the indicated antibodies. The degree of protein phosphorylation was calculated with ImageJ software and is presented under the blot. (G–K) Effects of DDX56-RNAi plasmids on SeV-triggered transcription of *IFNB1*, *TNFa*, *Il8*, *Rantes* and *Isg56* genes. The stable DDX56-knockdown 293T cells (4×10^5^) were left uninfected or infected with SeV for 12 h before qRT-PCR was performed. Shown are representative experiments of three independent experiments with mean±s.d. of three technical replicates. **P<0.01. Luc, luciferase; Coni, control siRNA.

We next investigated the function of endogenous DDX56 during SeV- or poly(I:C)-triggered type I IFN production. We constructed two RNAi plasmids for DDX56. The DDX56-RNAi#1 and DDX56-RNAi#2 RNAi plasmids could markedly reduce the expression of endogenous DDX56 in 293T and THP-1 cells (Fig. 2A; Fig. S2A). In reporter assays, knockdown of DDX56 potentiated SeV- or poly(I:C)-triggered activation of the IFN-β promoter and ISRE (Fig. 2B–E). Further experiments indicated that SeV-triggered phosphorylation of TBK1, IRF3, p65 (also known as RELA) and IkBa was markedly higher in DDX56-knockdown cells in comparison to what was seen in control cells (Fig. 2F). In real-time quantitative (q)RT-PCR experiments, we observed that SeV-triggered transcription of the *Ifnb1*, *Tnfa*, *Il8*, *Rantes* and *Isg56* genes was markedly increased in DDX56-knockdown 293T and THP-1 cells in comparison with that in control cells (Fig. 2G–K; Fig. S2B–F). Collectively, these results suggest that knockdown of DDX56 potentiates the virus-triggered induction of IFN-β.

### Deficiency of DDX56 potentiates virus-triggered induction of IFN-β

**Fig. 3.**
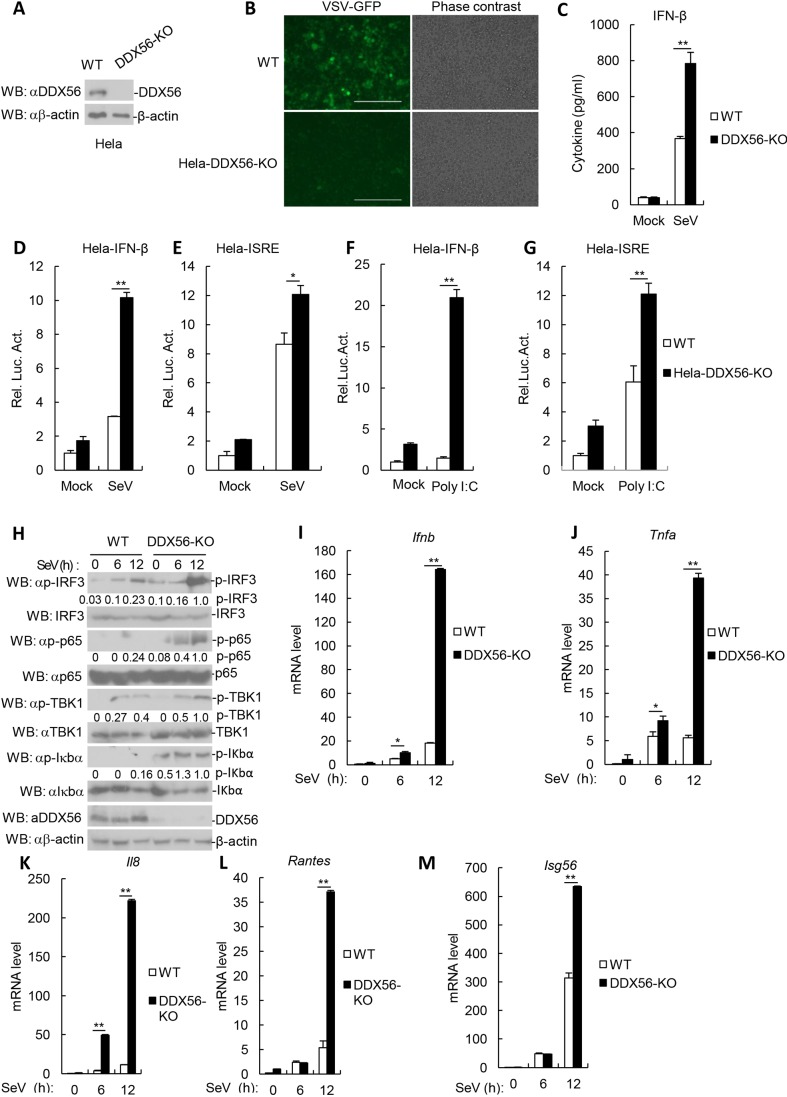
**Knockout of DDX56 potentiates RNA virus-triggered IFN-β signaling.** (A) DDX56 levels in the HeLa cells were analyzed by immunoblotting. (B) VSV replication in wild-type and DDX56- knockout (KO) HeLa cells. HeLa (5×10^4^) were infected by VSV–GFP (MOI 0.1) for 2 h and imaged by microscopy. Scale bar: 400 µm. (C) Effects of DDX56 deficiency on secretion of IFN-β induced by SeV in HeLa cells. DDX56-knockout HeLa cells were infected with SeV for 12 h. The culture medium was collected for quantification of the indicated cytokines by ELISA. The experiment shown is representative of three independent experiments with mean±s.d. of three technical replicates. **P<0.01. (D–G) Effects of DDX56 knockout on SeV- or poly(I:C)-induced activation of the IFN-β promoter and ISRE. DDX56-knockout HeLa cells (10^5^) were transfected with the IFN-β promoter or ISRE (100 ng). At 24 h after transfection, the cells were left uninfected or infected with SeV for 12 h or were treated or untreated with poly(I:C) (1 µg/ml) for 18 h before reporter assays were performed. Shown are representative experiments of three independent experiments with mean±s.d. of three technical replicates. *P<0.05, **P<0.01. (H) Effects of DDX56 deficiency on SeV-induced phosphorylation of TBK1, IRF3, p65 and IκBα. The DDX56-knockout HeLa cells were untreated or treated with SeV for the indicated times, and cell lysates were analyzed by immunoblotting with the indicated antibodies. The degree of protein phosphorylation was calculated by ImageJ software and is presented under the blot. (I–M) Effects of DDX56 deficiency on SeV-triggered transcription of *IFNB1* and *TNFa* genes. The DDX56-knockout HeLa cells (4×10^5^) were left uninfected or infected with SeV for 12 h before qRT-PCR was performed. Shown are representative experiments of three independent experiments with mean±s.d. of three technical replicates. *P<0.05, **P<0.01. WT, wild type; Luc, luciferase.

To further examine the roles of DDX56 in the antiviral innate immune response, we made DDX56- knockout HeLa cells through CRISPR/Cas9-mediated genome editing. Immunoblot analysis confirmed that DDX56 was undetectable in the DDX56-knockout HeLa cells (Fig. 3A). We observed that the replication of VSV was markedly reduced in DDX56-knockout HeLa cells compared with the wildtype cells (Fig. 3B). Additionally, ELISA experiments indicated that the levels of secreted IFN-β induced by SeV infection increased in DDX56-knockout cells in comparison to what was seen wild-type cells (Fig. 3C). In reporter assays, SeV- or poly(I:C)-triggered activation of the IFN-β promoter and ISRE was markedly higher in DDX56-knockout HeLa cells than in their wild-type counterparts (Fig. 3D–G). Moreover, SeV-triggered phosphorylation of TBK1, IRF3, p65 and IkBa were markedly higher in DDX56-knockout cells in comparison to wild-type cells (Fig. 3H). In qRT-PCR experiments, we observed that SeV-triggered transcription of the *Ifnb1*, *Tnfa*, *Il8*, *Rantes* and *Isg56* genes was markedly increased in DDX56-knockout HeLa cells in comparison with control cells and reconstitution of DDX56 into DDX56-knockout HeLa cells (Fig. 3I–M; Fig. S3A–E). As a control for our process, we determined that there were 11 potential off-target sites for the DDX56 CRISPR/Cas9 process through the Cas-OFFinder tool (Fig. S3F), but sequencing did not detect off-target editing (data not shown). Collectively, these results suggest that DDX56 plays a key role in the control of the IFN-β signaling pathway.

### DDX56 regulates virus-triggered signaling at the level of IRF3

**Fig. 4.**
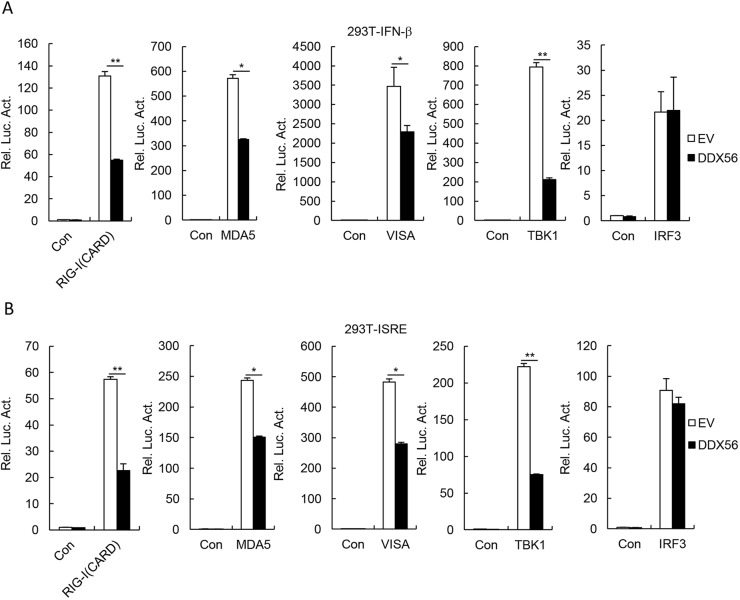
**DDX56-mediated virus-triggered signaling at the level of IRF3.** (A,B) Effects of DDX56 on the IFN-β promoter and ISRE activation by various signaling components. 293T cells (10^5^) were transfected with the IFN-β promoter (A) or ISRE (B) reporter (0.1 µg), and the expression plasmids for DDX56 and the indicated plasmids (0.1 µg each). Luciferase assays were performed 24 h after transfection. Shown are representative experiments of three independent experiments with mean±s.d. of three technical replicates. *P<0.05, **P<0.01. EV, empty vector; Luc, luciferase; Con, control.

Various components are involved in virus-triggered signaling pathways. In the reporter assays, DDX56 inhibited the IFN-β promoter and ISRE activation that is mediated by RIG-I (the CARD domain), MDA5, VISA and TBK1, but not IRF3 (Fig. 4A,B). These results suggest that DDX56 targets IRF3 or a signaling step upstream of IRF3.

### DDX56 interacts with IRF3

**Fig. 5.**
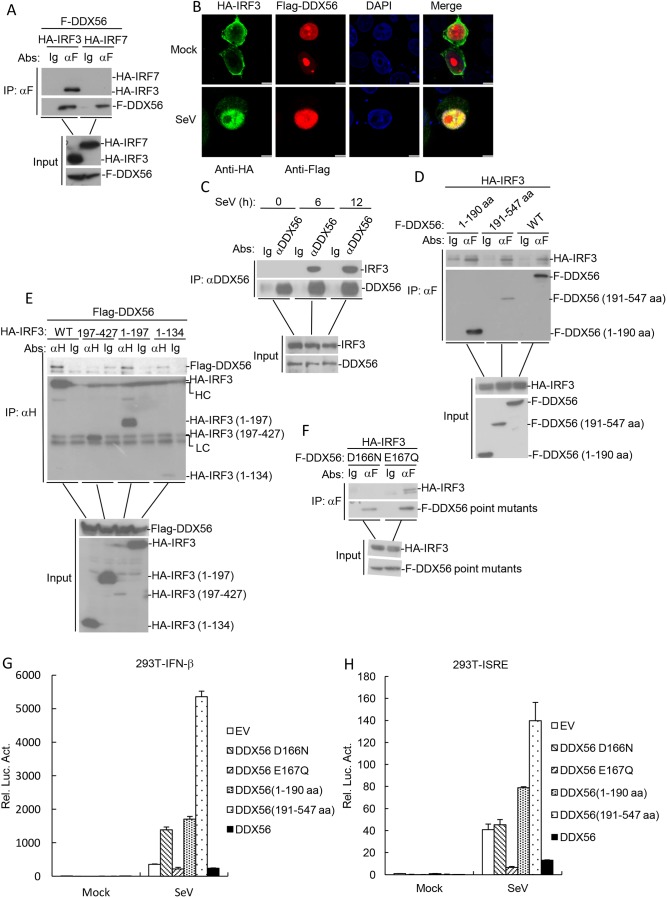
**DDX56 interacts with IRF3.** (A) DDX56 interacted with IRF3 but not with IRF7 in the overexpression system. 293T cells (2×10^6^) were transfected with the indicated plasmids (5 μg each). Co-immunoprecipitation and immunoblotting analyses were performed with the indicated antibodies. The expression of the transfected proteins was analyzed by immunoblotting with anti-HA or anti-Flag antibodies. (B) Colocalization of DDX56 with the IRF3. HeLa cells were transfected with HA–IRF3 (1 µg) and Flag–DDX56 (1 µg) plasmids. At 24 h after transfection, cells were left untreated or infected with SeV for 6 h before confocal microscopy. Scale bar: 5 µm. (C) Endogenous association between DDX56 and IRF3. 293T cells were left untreated or treated with SeV for the indicated times before co-immunoprecipitation and immunoblot analysis. (D) DDX56 mutants interact with IRF3. 293T cells (2×10^6^) were transfected with the indicated plasmids (5 µg each). Co-immunoprecipitations were performed with anti-Flag or control IgG. Immunoblotting analysis was performed with anti-HA antibody (upper panels). Expression levels of the proteins were analyzed by immunoblotting analysis of the lysates with anti-HA and anti-Flag antibodies (lower panels). (E) The 1–197 domain of IRF3 is required for its interaction with DDX56. Experiments were performed in a similar manner to those in C. (F) The D166 site of DDX56 is required for its interaction with IRF3. Experiments were performed in a similar manner to those in C. (G,H) Effects of DDX56, its mutants and its point mutants on the IFN-β promoter and ISRE activation. 293T cells (10^5^) were transfected with the ISRE (H) or IFN-β promoter (G) luciferase plasmids (0.1 μg) and the indicated expression plasmids (100 ng). At 24 h after transfection, cells were infected with or without SeV for 10 h before the luciferase assays were performed. The experiment shown is representative experiments of three independent experiments with mean±s.d. of three technical replicates. EV, empty vector; Luc, luciferase; LC, light chain; HC, heavy chain.

Since DDX56 targets IRF3 or an signaling step upstream of IRF3, we determined whether DDX56 is associated with IRF3. In transient transfection and co-immunoprecipitation experiments, DDX56 interacted with IRF3 but not IRF7 (Fig. 5A). In addition, confocal microscopy experiments indicated that DDX56 was colocalized with IRF3 after SeV infection in the cell nucleus (Fig. 5B). Furthermore, endogenous co-immunoprecipitation experiments indicated that DDX56 was associated with IRF3 in 293T cells, and this association was not affected following SeV infection (Fig. 5C). Interestingly, both DDX56 [amino acids (aa) 1–190] and DDX56 (aa 191–547) interact with IRF3 (Fig. 5D). Domain mapping experiments indicated that IRF3 (aa 1–197) is required for the interaction with DDX56 (Fig. 5E). Since a previous study had shown that the D166 and E167 sites of DDX56 play a role in its helicase activity (Pause and Sonenberg, 1992), we investigated whether D166 or E167 mediated the interaction between DDX56 and IRF3. We made DDX56 mutants in which the asparagine residue was mutated to aspartate or the glutamine residue were mutated to glutamate. As shown in Fig. 5F, mutation of D166N, but not E167Q, resulted in a loss of interaction with IRF3. In addition, DDX56E167Q and DDX56 inhibited SeV-triggered activation of the IFN-β promoter and ISRE, but not DDX56 (aa 1–190), DDX56 (aa 191–547) and DDX56 D166Q (Fig. 5G,H). Collectively, these results suggest that DDX56 interacts with IRF3, and the D166 site of DDX56 is important in its innate immune function.

### DDX56 suppresses the import of IRF3 into the nucleus

**Fig. 6.**
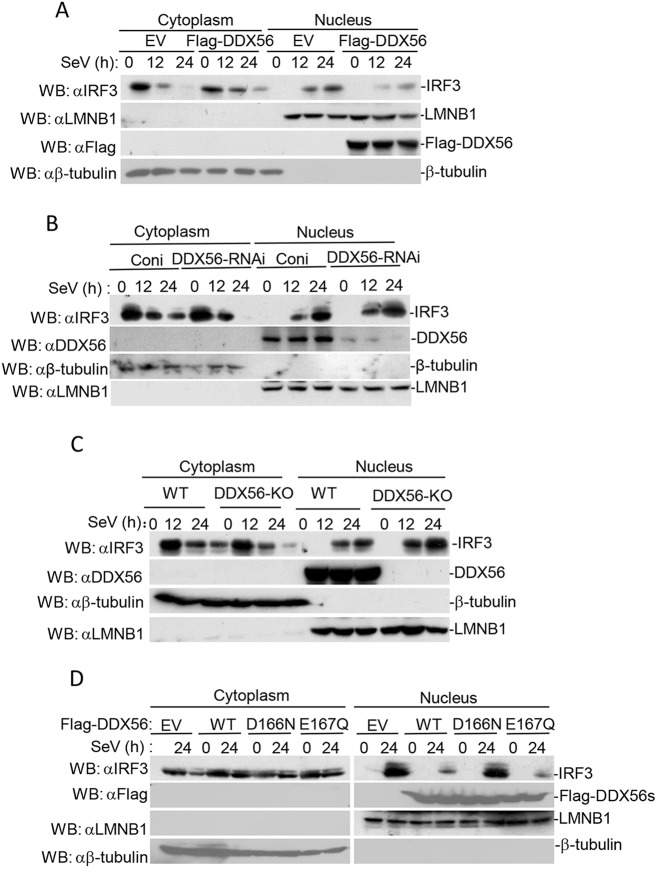
**DDX56 inhibits the nuclear translocation of IRF3.** (A) Overexpression of DDX56 inhibits nuclear translocation of IRF3. Stably transfected DDX56 293T cells (5×10^7^) were left untreated or treated with SeV for the indicated time. Cell fractionation was performed, and the cytoplasmic and nuclear proteins were prepared in 1 ml of homogenization buffer or nuclear extraction buffer, respectively, followed by co-immunoprecipitation and immunoblotting (WB) analysis. (B) Effects of DDX56 knockdown on IRF3 nuclear translocation. The DDX56-RNAi stable cells (5×10^7^) were left untreated or treated with SeV for the indicated time points. Experiments were performed similar to those in A. (C) DDX56 deficiency increases nuclear translocation of IRF3 in HeLa cells. Experiments were performed in a similar manner to those in A. (D) Effects of DDX56D166N and DDX56E167Q on the nuclear translocation of IRF3. Experiments were performed in a similar manner to those in A. EV, empty vector; WT, wild type; Coni, control siRNA.

Upon virus infection, IRF3 undergoes C-terminal phosphorylation and dimerization and then translocates into the nucleus, subsequently forming a complex with the coactivators CBP/p300, and binds to the positive regulator domain (PRD) and PRD III-like elements (PRD-LEs) of the IFN-a/b promoters and ISRE sequences of targeted genes, including that encoding the cytokine RANTES and IP-10 (Lin et al., 1998; Servant et al., 2002; Yoneyama et al., 1998). Because IRF3 translocates into the nucleus, which is a key step of IRF3 activating the transcription of type I IFN genes, we examined whether DDX56 is involved in the nuclear translocation of IRF3. In subcellular fractionation and immunoblot experiments, the import of IRF3 into the nucleus after challenge with SeV was significantly inhibited in DDX56-overexpressing 293T cells relative to that in control cells (Fig. 6A). We obtained the opposite results for cells in which DDX56 was knocked down in 293T cells or knocked out in HeLa cells (Fig. 6B,C). In our above experiments, we demonstrated that DDX56E167Q inhibited the SeV-triggered activation of the IFN-β promoter and ISRE (Fig. 5G,H). We therefore determined whether DDX56E167Q had an effect on the import of IRF3 into the nucleus. We found that nuclear IRF3 was significantly decreased in DDX56 and DDX56E167Q, but not DDX56D166N overexpression cells, compared to levels in control cells (Fig. 6D). These data demonstrate a critical role for DDX56 and its D166 site in the import of IRF3 into the nucleus.

### DDX56 interferes with the interaction between IRF3 and IPO5

**Fig. 7.**
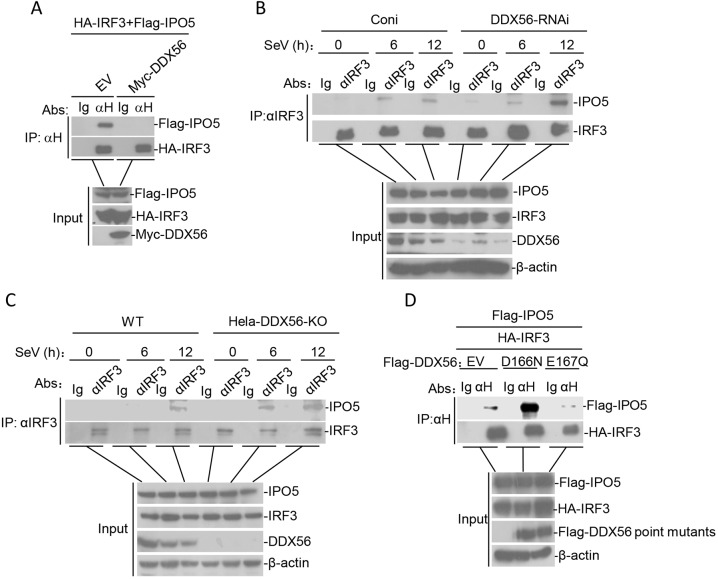
**DDX56 disrupts the IRF3–IPO5 interaction.** (A) Effects of DDX56 overexpression on the IRF3–IPO5 interaction. 293T cells (2×10^6^) were transfected with the indicated plasmids (5 µg each). Co-immunoprecipitations (IP) were performed with anti-HA antibody or control IgG. Immunoblot analysis was performed with anti-HA and anti-Flag antibodies (upper panels). Expression levels of the proteins were analyzed by immunoblot analysis of the lysates with anti-HA and anti-Flag (lower panels). (B) Effects of DDX56 knockdown on the IRF3–IPO5 interaction in 293T cells. The DDX56-RNAi stable 293T cells (5×10^7^) were left untreated or treated with SeV for the indicated times. Co-immunoprecipitations were performed with anti-IRF3 antibody or control IgG. (C) Effects of DDX56 deficiency on the IRF3–IPO5 interaction in HeLa cells. Experiments were performed in a similar manner to those in B. (D) DDX56D166N does not affect the IRF3–IPO5 interaction. The 293T cells (3×10^6^) were transfected with the indicated plasmids together with DDX56D166N or DDX56E167Q expression plasmids (5 µg). Co-immunoprecipitation and immunoblotting were performed with the indicated antibodies. EV, empty vector; H, HA tag.

Since previous studies have shown that IRF3 nuclear translocation is associated with IPO5 (also known as importin-β3) (Song et al., 2016), we investigated whether DDX56 interferes with the translocation of IRF3 into the nucleus by inhibiting the interaction between IRF3 and IPO5. In transient transfection and co-immunoprecipitation experiments, the overexpression of DDX56 interferes with the interaction between IRF3 and IPO5 (Fig. 7A). In contrast, the interaction between IRF3 and IPO5 was significantly increased in DDX56-knockdown or knockout cells relative to that in control cells (Fig. 7B,C). Furthermore, we found that DDX56D166N, but not DDX56E167Q, interferes with the interaction between IRF3 and IPO5 (Fig. 7D). These results showed that DDX56 and its D166 site play an important role in interfering with the interaction between IRF3 and IPO5.

## DISCUSSION

The nucleolar RNA helicase DDX56 is a member of the DEAD box RNA helicases (Zirwes et al., 2000). However, the functions of DDX56 remain unclear. Here, we have reported a previously unknown function for DDX56 that is associated with host defense against viral invasion. We demonstrated that DDX56 functions as a negative regulator of virus-triggered IFN-β induction by reducing nuclear IRF3, the master controller of the production of type I interferon. In addition, we found that DDX56 inhibited the phosphorylation of TBK1, IRF3, p65 and IκBa. We speculated that DDX56 could be involved in the NF-κB signaling pathway to inhibit type I interferon, but the mechanism requires further elucidation. Taken together, our results provide a new and direct link between DDX56 and antiviral innate immunity.

Previous studies have reported that the DEAD box motif is highly conserved in a subgroup of RNA helicases (Schmid and Linder, 1992) and mutagenic analyses revealed that the substitution of asparagine (N) for aspartate (D) or glutamine (Q) for glutamate (E) results in complete loss of helicase activity (Pause and Sonenberg, 1992). The two DEAD box mutants (D166N and E167Q) of DDX56 lack helicase function (Xu and Hobman, 2012). In our study, we found that D166, but not E167 of DDX56, plays a role in regulating innate immunity. Our study indicated that site D166 and site E167 have distinct functions, but the mechanism requires further elucidation.

In eukaryotes, there are total of 59 DExD/H helicases, which have been grouped into the RIG-1-like, DEAH/RHA, DEAD box and Ski2-like subfamilies (Linder, 2006). The role played by DExD/H helicases in antiviral innate immune responses may be broader than previously thought. For example, Dicer, another RIGI-like DExD/H helicase, was found to sense viral nucleic acids in the *Drosophila* innate immune system (Deddouche et al., 2008). Additionally the DDX1–DDX21–DHX36 complex represents a dsRNA sensor that uses the TRIF pathway to activate type I IFN responses in the cytosol of mouse dendritic cells (Zhang et al., 2011). Previous studies have shown that many DExD/H helicases activate type I IFN responses, including DDX58, DDX3, DHX29, DHX36 and DDX60 (Desmet and Ishii, 2012; Loo and Gale, 2011; Sugimoto et al., 2014; Yoo et al., 2014). Only a few DExD/H helicases inhibit type I IFN responses, such as DDX24 and DDX46 (Ma et al., 2013; Zheng et al., 2017). DExH/D helicases are broadly involved in many RNA-related processes such as transcription, translation, ribosome biogenesis, RNA transportation and RNA modifications (Lüking et al., 1998; Silverman et al., 2003; de la Cruz et al., 1999). Since DDX56 is a protein that is poorly studied, whether its function could be linked to its RNA-binding activity will need to be considered in the future. Here, we report that an IFN-inducible helicase, referred to as DDX56, is able to negatively regulate the RIG-I-like receptor (RLR) signaling pathway by disrupting the assembly of IRF3–IPO5 complex to suppress the import of IRF3 into the nucleus.

In summary, we have further characterized a new IFN-inducible DExD/H helicase, DDX56, that is involved in a negative-feedback role to regulate the RLR pathway and type I IFN production. A further understating of these processes may shed light on the causes of infectious disease, and possibly inflammatory disorders, that involve enhanced innate immune gene activity.

## MATERIALS AND METHODS

### Cells, reagents and antibodies

Human embryonic kidney 293T (293T), HeLa and THP-1 cells were cultured with high-glucose DMEM (Hyclone) medium plus 10% heat-inactivated FBS (Hyclone) and were supplemented with antibiotics (100 unit/ml penicillin, 100 mg/ml streptomycin, Gibco). The nuclear-cytosol extraction kit was purchased from ApplyGen. Mouse monoclonal antibodies (mAbs) against Flag (Sigma-Aldrich, F1804, 1:1000) and β-actin (Sigma-Aldrich, A5441, 1:500), hemagglutinin (HA) (Convance, MMS-101R-1000, 1:1000) and β-tubulin (Invitrogen, 32-2600, 1:1000); rabbit polyclonal antibodies against LMNB1 (Proteintech, 12987-1-AP, 1:1000), IPO5 (Abcam, ab187175, 1:1000), DDX56 (Abcam, ab97648, 1:1000), TBK1 (Abcam, ab40676, 1:1000), Flag (Sigma-Aldrich, F7425, 1:1000) and p-TBK1(S172) (Abcam, ab109272, 1:1000); IRF3 (Cell Signaling Technology, 4302, 1:1000), and p-IRF3(S396) (Cell Signaling Technology, 4947, 1:1000); and polyinosinic-polycytidylic acid [poly(I:C)] (InvivoGen) were purchased from the indicated companies. Sendai virus (SeV) and VSV-GFP were used as previously described (Zhong et al., 2008).

### Constructs

ISRE, the IFN-β promoter luciferase reporter plasmids and mammalian expression plasmids for HA-tagged RIG-I (CARD), MDA5, VISA, TBK1, IRF3, and IRF3-5D were constructed as previously described (Diao et al., 2007; Lei et al., 2010; Zhong et al., 2010). The mammalian expression plasmids for Flag- or Myc-tagged DDX56, its mutants and point mutants, HA-tagged IRF3 truncations were constructed with standard molecular biology techniques. The pSuper.retro RNAi and pMSCV plasmids were provided by Hongbing Shu (Wuhan University, China).

### MTT assay

293T cells were trypsinized and seeded into 96-well plates at a density of ∼4000 cells per well. After 24 h, adherent cells were transfected with empty vector or Flag–DDX56 plasmids (0 µg, 0.25 µg or 0.5 µg); non-transfected 293T cells served as the control group. During the following 2 days, MTT reagent [3-(4,5-dimethylthiazol-2-yl)-2,5-diphenyltetrazolium bromide; Sigma-Aldrich] was added to the cells (20 µl (5 mg/ml) per well), and cells were then incubated for 4 h at 37°C. The cell medium was removed, and 150 µl of DMSO was added to each well followed by gentle shaking of the plates to dissolve the formazan crystals. The optical density (OD) was then measured using a microplate reader at 490 nm.

### Apoptosis assay by flow cytometry

Apoptosis was analyzed to identify and quantify apoptotic cells with a Annexin V-FITC/PI apoptosis detection kit (Beyotime Institute of Biotechnology, Haimen, China). The 293T cells were seeded in a six-well plate for 12 h and then transfected with empty vector or Flag–DDX56 plasmids (1 µg) for 24 h. Cells were collected by trypsinization, washed twice with PBS and centrifuged at 500 ***g*** at room temperature for 5 min. The cells were then suspended in 500 μl PBS and incubated with 5 μl Annexin V–FITC (Annexin V–FITC Apoptosis Detection Kit; Beyotime Institute of Biotechnology, Haimen, China) and 10 μl (20 μg/ml) propidium iodide (PI) solution (Beyotime Institute of Biotechnology) at room temperature for 20 min in the dark. The samples were then assessed using a flow cytometer.

### Fluorescence confocal microscopy

293T cells were transfected with the indicated HA–IRF3 (1 µg) and Flag–DDX56 (1 µg) plasmids with Lipofectamine 2000 (Invitrogen). At 24 h after transfection, the cells were infected with SeV for 6 h and then fixed with 4% paraformaldehyde for 10 min at room temperature and permeabilized with 0.1% Triton X-100 for 15 min. The cells were then incubated with anti-Flag tag rabbit polyclonal antibody or anti-HA tag mouse monoclonal antibody. The cells were then incubated with goat anti-mouse IgG (whole molecule)-fluorescein isothiocyanate antibody (Sigma-Aldrich, F2012, 1:1000) and goat anti-rabbit IgG (whole molecule)-tetramethyl rhodamine isocyanate (TRITC) antibody (Sigma-Aldrich, T6778, 1:1000). Cells were stained with 4′,6-diamidino-2-phenylindole (DAPI) for 15 min and examined with a Leica SP2 confocal system (Leica Microsystems).

### Isolation of cytoplasmic and nuclear fraction

293T and HeLa cells, either infected with SeV or left uninfected, were washed with PBS and lysed with 40 strokes of a dounce homogenizer in 1 ml of cytosol extraction reagent (ApplyGen). The homogenate was centrifuged at 500 ***g*** for 5 min. The supernatant was saved as cytoplasm. The pellet was washed twice with 500 ml of nuclear extraction reagent (ApplyGen) and centrifuged twice at 4000 ***g*** for 5 min to precipitate the nuclei. The precipitates were analyzed by standard immunoblotting procedures.

### CRISPR-Cas9

The protocols for genome engineering using the CRISPR-Cas9 system were as previously described (Chen et al., 2016). The pGL-U6-gRNA and pST1374-Cas9-D10A plasmids were provided by Hongbing Shu. The guide RNA sequence (DDX56, 5′-GACAGGTCCGGTGGTAGAAC-3′) was designed using the online tool by F. Zhang (http://crispr.mit.edu/). The pGL-U6-gRNA plasmid (with sgRNA cloned) and pST1374-Cas9-D10A plasmids was co-transfected into HeLa cells for 24 h, followed by selection with puromycin (2 μg ml^−1^, Life Technologies) for 2 weeks. Protein expression was determined by immunoblot analysis.

### Transfection and reporter gene assays

293T cells or HeLa cells (1×10^5^) were seeded into 48-well plates and transfected the following day via a a standard calcium phosphate precipitation method. Empty control plasmid was added to ensure that each transfection received the same amount of total DNA. To normalize for transfection efficiency, 10 ng of pRL-TK *Renilla* luciferase reporter plasmid, 100 ng of either ISRE or the IFN-β promoter and 100 ng of the indicated plasmid were added to each transfection. The luciferase assays were performed with a dual-specific luciferase assay kit (Promega). Firefly luciferase activities were normalized against the *Renilla* luciferase activities.

### Quantitative real-time PCR

Total RNA was isolated and reversed transcribed to cDNA for quantitative real-time PCR analysis to measure the mRNA levels of the tested genes. GAPDH was used as a reference gene. The human gene specific primer sequences were as follows: *GAPDH*, 5′-GAGTCAACGGATTTGGTCGT-3′ (forward), 5′-GACAAGCTTCCCGTTCTCAG-3′ (reverse); *IFNb*, 5′-TTGTTGAGAACCTCCTGGCT-3′ (forward), 5′-TGACTATGGTCCAGGCACAG-3′ (reverse); *TNFa*, 5′-GCCGCATCGCCGTCTCCTAC-3′ (forward), 5′-CCTCAGCCCCCTCTGGGGTC-3′ (reverse); *Il8*, 5′-GAGAGTGATTGAGAGTGGACCAC-3′ (forward), 5′-CACAACCCTCTGCACCCAGTTT-3′ (reverse); *Rantes*, 5′-GGCAGCCCTCGCTGTCATCC-3′ (forward), 5′-GCAGCAGGGTGTGGTGTCCG-3′ (reverse); and *Isg56*, 5′-TCATCAGGTCAAGGATAGTC-3′ (forward), 5′-CCACACTGTATTTGGTGTCTAGG-3′ (reverse).

### RNAi experiments

Double-stranded oligonucleotides corresponding to the target sequences were cloned into the pSuper-Retro RNAi plasmid. Small interfering RNAs corresponding to the same target sequences were purchased from GenePharma. The following sequences were targeted for human DDX56 cDNA: #1, 5′-GCAAGACAGCCTGAAACTTCG-3′; #2, 5′-GCTCTTTGTCAACACTCTAGA-3′.

### Co-immunoprecipitation and immunoblotting analysis

For co-immunoprecipitation assays, cells were collected 24 h after transfection and then lysed in 0.5% Triton X-100 buffer (20 mM Tris-HCl pH 7.5, 150 mM NaCl, 0.5% Triton X-100, 1 mM EDTA, 10% glycerol, 10 mg/ml aprotinin, 10 mg/ml leupeptin and 1 mM phenylmethylsulfonyl fluoride) on ice for 45 min, sonicated briefly, and the clarified supernatants were then incubated with anti-Flag or anti-HA antibody overnight at 4°C, followed by further incubation with protein A/G beads (Pierce) for 2–4 h. The immune complexes were washed with lysis buffer three times and subjected to immunoblot analysis with the indicated antibodies. For endogenous immunoprecipitation, 293T or HeLa cells were lysed with 1% NP-40 buffer, sonicated briefly, and the supernatants were incubated with DDX56 or IRF3 antibody or control IgG overnight at 4°C, followed by further incubation with protein A/G beads (Pierce) for 2–4 h. Immunoblotting was carried out by standard procedures.

### RNAi or DDX56-transduced stable 293T and THP-1 cells

Control or DDX56-RNAi/DDX56 retroviral plasmids were co-transfected with packaging plasmids (pGAG-Pol and pVSV-G) into 293T or THP-1 cells. At 24 h after transfection, the cell culture medium was replaced with new medium without antibiotics, and the cells were incubated for 24 h. The 293T or THP-1 cells were then infected with recombinant virus-containing medium in the presence of polybrene (6 µg/ml) and were selected with puromycin (1 µg/ml) for 1 month before additional experimentation.

### Statistical analysis

The significance of differences between samples was assessed using an unpaired two-tailed Student's *t*-test. The variance was estimated by calculating the standard deviation (s.d.) and is represented by error bars. All experiments were performed independently at least three times, with a representative experiment being shown. *P<0.05, **P<0.01.

## Supplementary Material

Supplementary information
