# Correction: DDX56 inhibits type I interferon by disrupting assembly of IRF3–IPO5 to inhibit IRF3 nucleus import

**DOI:** 10.1242/jcs.244681

**Published:** 2020-02-20

**Authors:** Dan Li, Shaozu Fu, Zhengqian Wu, Wenping Yang, Yi Ru, Hongbing Shu, Xiangtao Liu, Haixue Zheng

There was an error published in J. Cell Sci. (2020) 133, jcs230409 (doi: 10.1242/jcs.230409).

On p. 3, in the section entitled ‘DDX56 interacts with IRF3’, the authors mistakenly said that endogenous co-immunoprecipitation experiments indicated that DDX56 association with IRF3 in 293T cells was not affected following SeV infection (see Fig. 5C).

The correct text is: ‘Furthermore, endogenous co-immunoprecipitation experiments indicated that DDX56 was associated with IRF3 in 293T cells, and this association was increased following SeV infection (Fig. 5C).’

The authors apologise to readers for any confusion that this error might have caused. Both the online full-text and PDF versions of the article have been updated.

