# Supplementary material for: DDX56 inhibits type I interferon by disrupting assembly of IRF3–IPO5 to inhibit IRF3 nucleus import
Source: J Cell Sci. 2019 Jul 24;133(5):jcs230409. doi: 10.1242/jcs.230409 (PMC6899003; doi:10.1242/jcs.230409)
Supplement: Supplementary information [file joces-133-230409-s1.pdf]

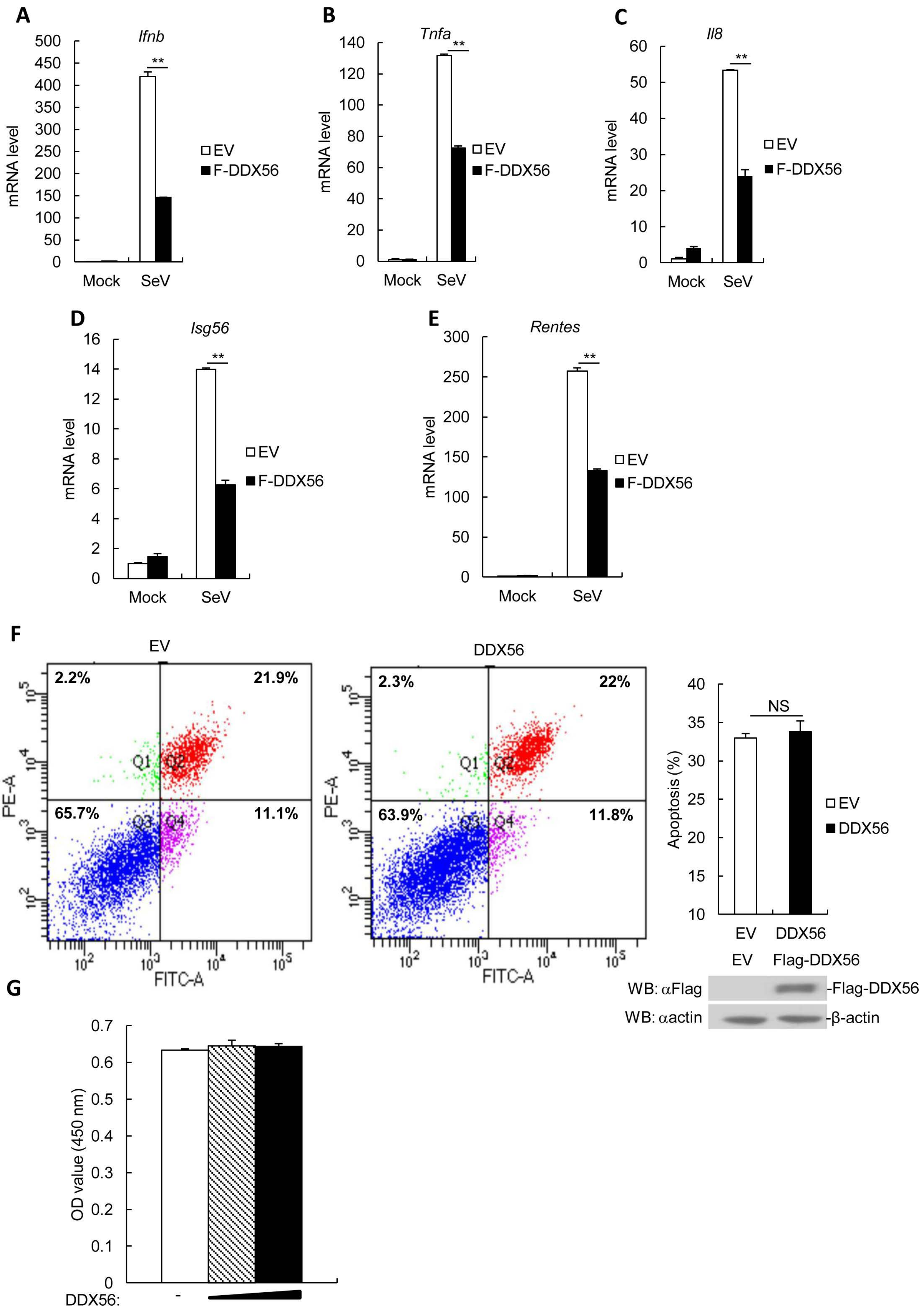

Journal of Cell Science • Supplementary information

Figure S1. The effects of DDX56 overexpression on transcription of antiviral genes in THP-1 cells. (A-E) The DDX56-overexpression stable THP-1 cells (4×10^5) were left uninfected or infected with SeV for 12 h before Q–PCR was performed. Shown are representative experiments of three independent experiments with mean±s.d. of three technical replicates. **$P<0.01$. (F) Effects of DDX56 on 293T cells apoptosis. 293T cells were transfected with empty vector or Flag-DDX56 plasmids (1 μg) for 24 h. The cellular apoptosis rate was measured by flow cytometry. Western blotting was used to analyze protein expression of DDX56. (G) Effects of DDX56 on 293T cells viability. 293T cells were transfected with empty vector or Flag-DDX56 plasmids for 24 h. Cells viability was measured by MTT. Shown are representative experiments of three independent experiments with mean±s.d. of three technical replicates. NS, not significant. EV, empty vector.

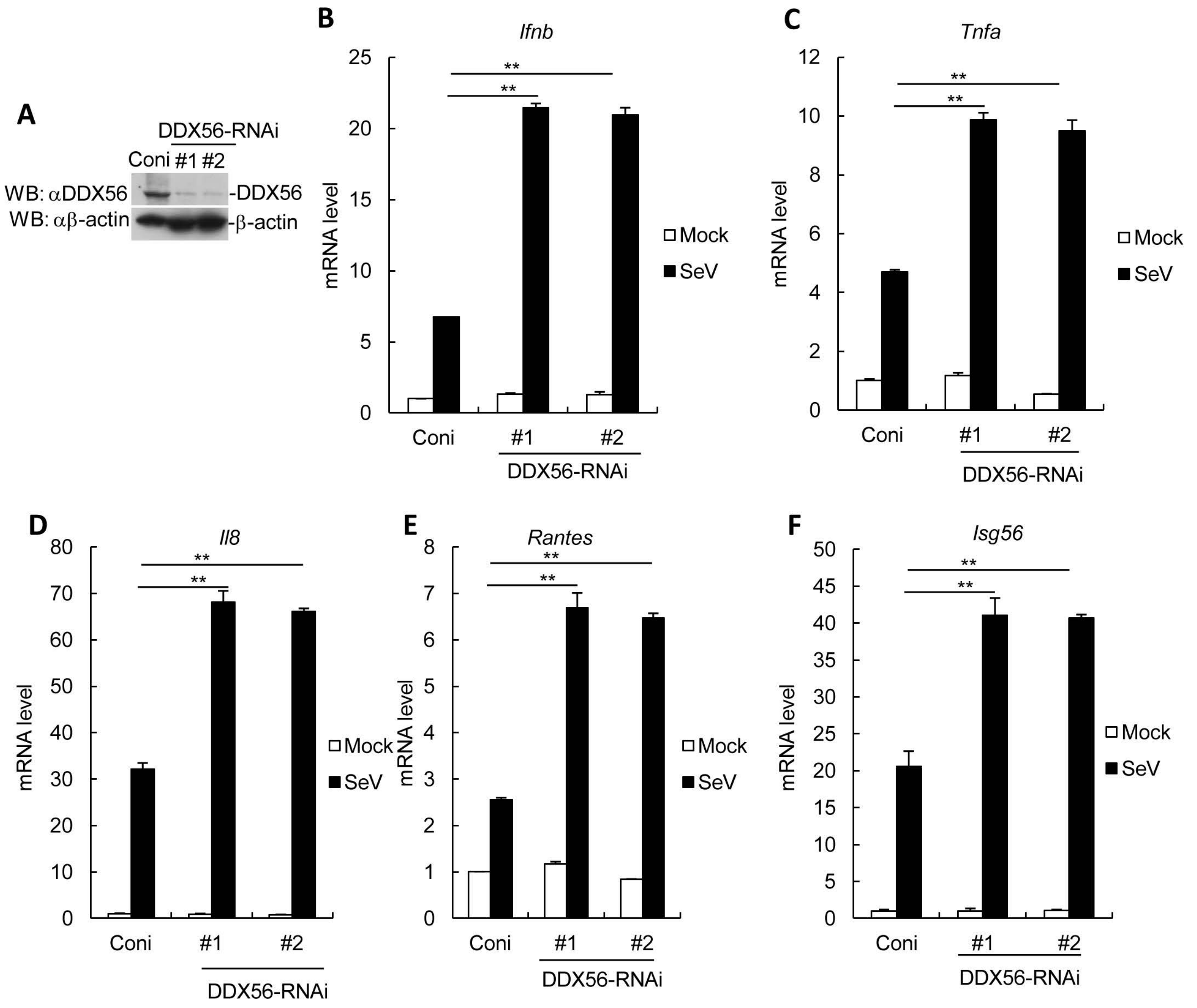

Figure S2. The effects of DDX56 knockdown on transcription of antiviral genes in THP-1 cells. (A) Immunoblot analysis of DDX56 protein levels in DDX56-overexpression stable THP-1 cells. (B-F) The DDX56-knockdown stable THP-1 cells (4×10^5) were left uninfected or infected with SeV for 12 h before Q–PCR was performed. Shown are representative experiments of three independent experiments with mean±s.d. of three technical replicates. $^{**}P$<0.01. EV, empty vector.

Journal of Cell Science • Supplementary information

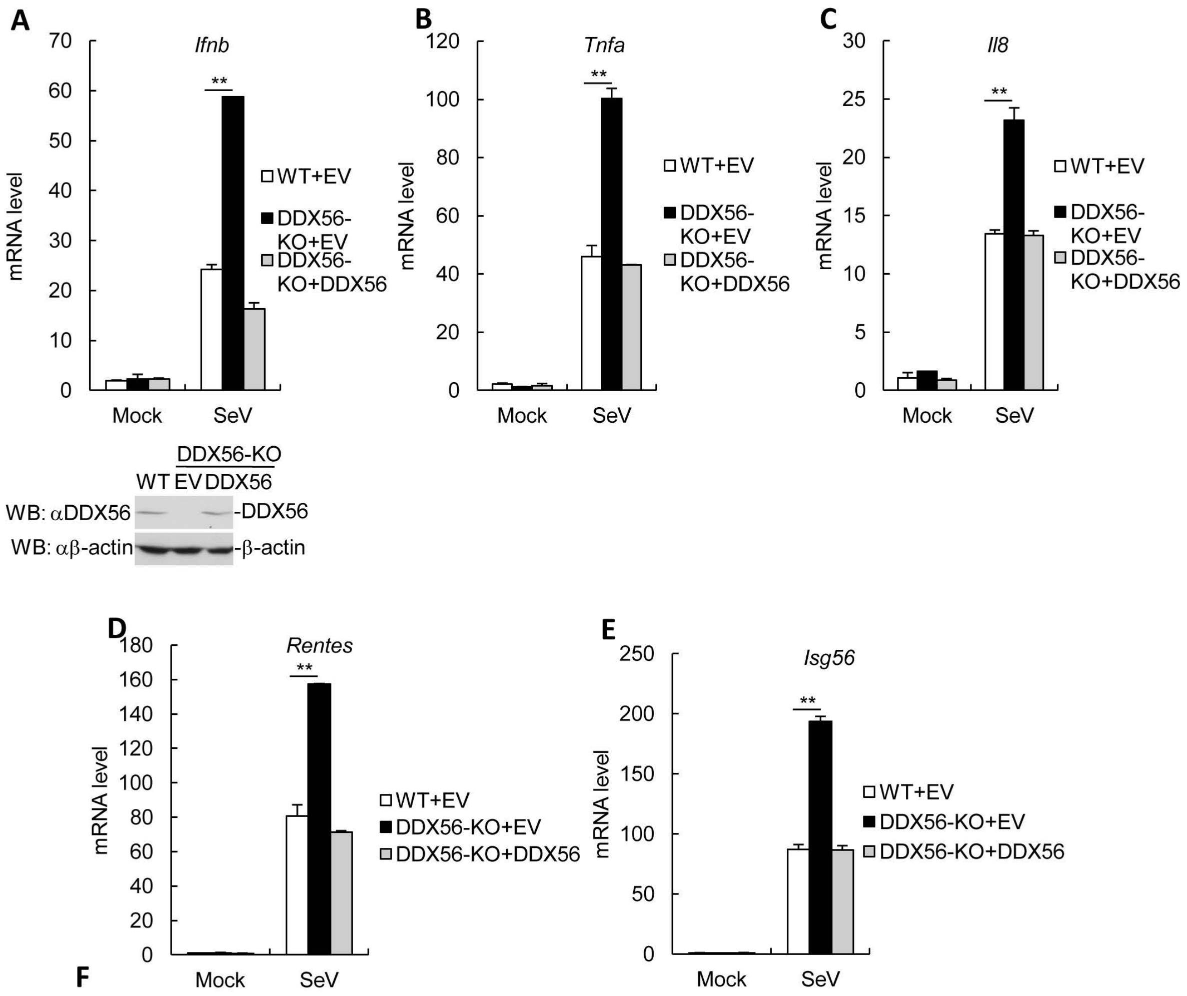

F

Potential off-target sites of the DDX56	Sequencing primers
GACAGGTCCTGTTGAAGAACAGG	F:GGGCCAAACTGCCGACATAA<br>R:CCTCCAGGGCTACTAACCGA
TGAAGGTCCGGAGGTAGAACCGG	F:GCATATCTGTCTCTCCCCAA<br>R:AGGAATGGTCATGTGACCTGG
AACAGGGCCGGGGCTAGAACAGG	F:CAAACCATGCCCCATCCTCT<br>R:CTCCCAGGGCCATGTGTAAG
AACAGGTGCAGTGGCAGAACTGG	F:TTTTCATGACCCAATGCGGC<br>R:TGCGTTTGCTGTCCATGTTG
GTTACGTCCGTTGGTAGAACTGG	F:GCCTTAAAAGAGTTGGCCTGAG<br>R:TGTGGGACCCCCAATGAATC
GGCAGGGACGGTGGGAGAACTGG	F:GGAACTTACCGTCTACCGGG<br>R:GCCCAGGGTTTCTCCAAGTC
GGCAGGGCAGGAGGTAGAACCGG	F:GGAGAAAACTCTGGCCCTCC<br>R:CACCCGGCGTTTCCTTATCA
GAAAGGGCAGCTGGTAGAACAGG	F:ATCAGCAGCCACCTGACATT<br>R:GCAGTTAACCGGGCGTGAG
GAAAGGTCGGGTATTAGAACTGG	F:GGCATTGTGCTACCCAGAATAG<br>R:CTTCAGCTCCTGCTCGTACAT
GAGAGGTCCTGGGATAGAACAGG	F:GCTAATCCTGTTAGCGTGGC<br>R:TGGTCTGGGGAATAGGGATGG
GACAGGTGGTGTGGGAGAACAGG	F:GATCATTAGCCATCAGCCCCC<br>R:CATGCCTGTGAAAGAGAAGCTC

Figure S3. (A-E) Effects of reconstitution of DDX56 on transcription of Ifnb, Tnfa, Il8, Rantens and Isg56 induced by SeV in DDX56-knockout Hela cells. The reconstituted cells were left uninfected or infected with SeV for 12 h before Q-PCR. Shown are representative experiments of three independent experiments with mean±s.d. of three technical replicates. ***P*<0.01. (F) The potential off-target sites of DDX56 and sequencing primers. EV, empty vector; KO, knockout; WT, wild type.
